# Action of Clathrodin and Analogues on Voltage-Gated Sodium Channels

**DOI:** 10.3390/md12042132

**Published:** 2014-04-04

**Authors:** Steve Peigneur, Aleš Žula, Nace Zidar, Fiona Chan-Porter, Robert Kirby, David Madge, Janez Ilaš, Danijel Kikelj, Jan Tytgat

**Affiliations:** 1 Catholic University Leuven (KULeuven), Toxicology and Pharmacology, Herestraat 49-Box 922, 3000 Leuven, Belgium; E-Mail: steve.peigneur@pharm.kuleuven.be; 2 University of Ljubljana, Faculty of Pharmacy, Aškerčeva 7, 1000 Ljubljana, Slovenia; E-Mails: ales.zula@ffa.uni-lj.si (A.Ž.); nace.zidar@ffa.uni-lj.si (N.Z.); janez.ilas@ffa.uni-lj.si (J.I.); danijel.kikelj@ffa.uni-lj.si (D.K.); 3 Xention Ltd., Iconix Park, London Road, Pampisford, Cambridge CB22 3EG, UK; E-Mails: fionachan31@yahoo.co.uk (F.C.-P.); robert.kirby@xention.com (R.K.); david.madge@xention.com (D.M.)

**Keywords:** clathrodin, oroidin, hymenidin, voltage-gated sodium channels, sodium channel modulator, sponge

## Abstract

Clathrodin is a marine alkaloid and believed to be a modulator of voltage-gated sodium (Na_V_ ) channels. Since there is an urgent need for small molecule Na_V_ channel ligands as novel therapeutics, clathrodin could represent an interesting lead compound. Therefore, clathrodin was reinvestigated for its potency and Na_V_ channel subtype selectivity. Clathrodin and its synthetic analogues were subjected to screening on a broad range of Na_V_ channel isoforms, both in voltage clamp and patch clamp conditions. Even though clathrodin was not found to exert any activity, some analogues were capable of modulating the Na_V_ channels, hereby validating the pyrrole-2-aminoimidazole alkaloid structure as a core structure for future small molecule-based Na_V_ channel modulators.

## 1. Introduction

**Figure 1:**
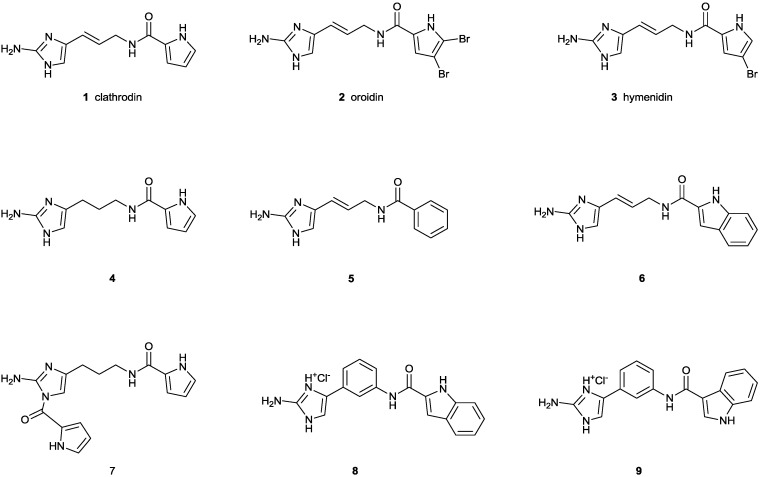
Structures of clathrodin (**1**), oroidin (**2**), hymenidin (**3**), dihydroclathrodin (**4**) and synthetic Analogues **5**, **6**, **7**, **8** and **9** are shown.

Marine organisms, such as sea anemones, cone snails, fish, algae and sponges, among others, are known to produce peptide and non-peptide toxins targeting ion channels. The majority of marine toxins have been characterized to act upon voltage-gated sodium (Na_V_) channels. These molecules exhibit their toxicity by either physically inhibiting the sodium ion flow through the channel or by modifying the kinetics of channel gating [1]. Clathrodin (**1**) is a 2-aminoimidazole alkaloid containing an unsubstituted pyrrole 2-carboxamide moiety, isolated from the Caribbean sea sponge, *Agelas clathrodes*, which is structurally related to its 2,3-dibromopyrrole analogue, oroidin, from the sponge, *Agelas oroides*, and 2-bromopyrrole analogue hymenidin from the sponge, *Hymeniacidon* sp. [2,3,4]. Clathrodin was shown, in experiments performed in cells isolated from chick embryo sympathetic ganglia using the whole cell configuration of the patch clamp technique, to possess neurotoxic activity. It decreased the average maximum amplitudes of isolated inward sodium currents by 30%. Electrophysiological experiments indicated that clathrodin had no effect on the voltage dependence of current activation. However, the voltage dependence of current inactivation was shifted towards more positive potentials, and the V_1/2_ of inactivation was changed by 14 mV, while no alteration of the time for current reactivation was observed. Clathrodin thus appeared to be a new sodium channel neurotoxin influencing sodium channel ionic conductance [5]. The exact site of interaction of clathrodin with the Na_V_ channel, as well as its subtype selectivity remained unknown [1]. Despite the fact that in the last 15 years, major progress has been made in understanding the structure and physiology of Na_V_ channels, together with an intensive quest for small molecules targeting Na_V_ channels as novel lead compounds for drug discovery [6,7,8], no further investigations on these three marine alkaloids have been reported. Aiming at using clathrodin as a possible lead for the design of Na_V_ channels modifiers with therapeutic potential, we decided to reinvestigate the effect of clathrodin and to determine its selectivity profile for different Na_V_ channel isoforms. This was done by combining the two-electrode voltage-clamp expression system with a high-throughput QPatch screening system. Additionally, with the aim of exploring preliminary structure activity relationship, we included in this study also the brominated analogues, oroidin (**2**) and hymenidin (**3**), as well as dihydroclathrodin (**4**), possessing a saturated linker between the 2-aminoimidazole core and the pyrrole-carboxamide moiety, two compounds in which the pyrrole is replaced by a phenyl (**5**) or the indole ring (**6**) and dihydroclathrodin derivative **7**, containing an additional dipyrrole ring attached to the 2-aminoimidazole core. Furthermore, conformationally restricted clathrodin analogues, **8** and **9**, containing a 1,3-phenylene linker between the 2-aminoimidazole core and the indole-carboxamide moieties were included in the study (Figure 1).

## 2. Results and Discussion

### 2.1. Results

**Figure 2:**
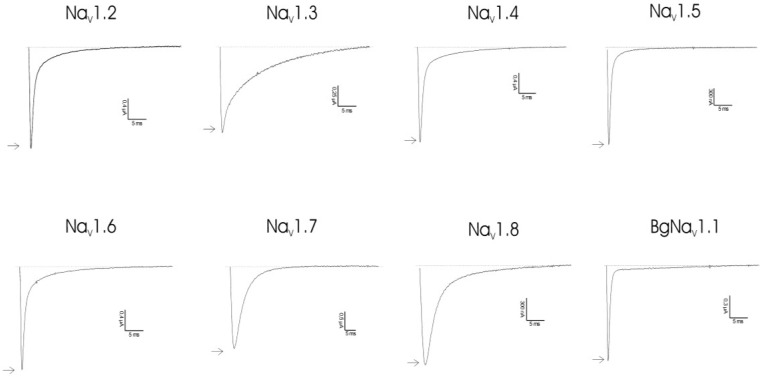
Activity profile of clathrodin on several Na_V_ channel isoforms. Representative whole-cell current traces in control and compound conditions are shown. The dotted line indicates the zero-current level. The arrow marks steady-state current traces after the application of 10 μM of clathrodin. The traces shown are representative traces of at least three independent experiments (*n* ≥ 3).

**Figure 3:**
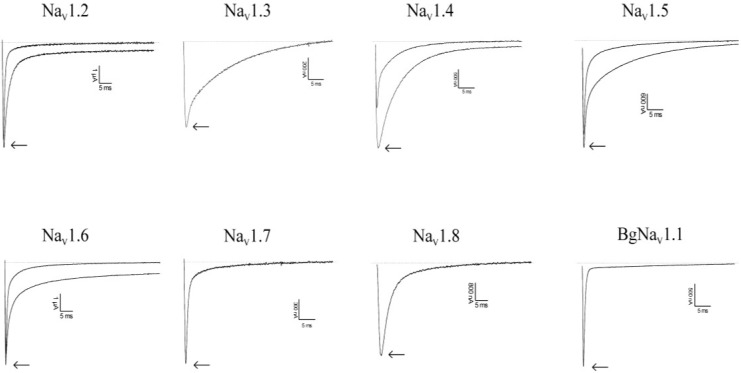
Activity profile of Compound **8** on several Na_V_ channel isoforms. Representative whole-cell current traces in control and compound conditions are shown. The dotted line indicates the zero-current level. The arrow marks steady-state current traces after the application of 10 μM of Compound **8**. The traces shown are representative traces of at least three independent experiments (*n* ≥ 3).

**Figure 4:**
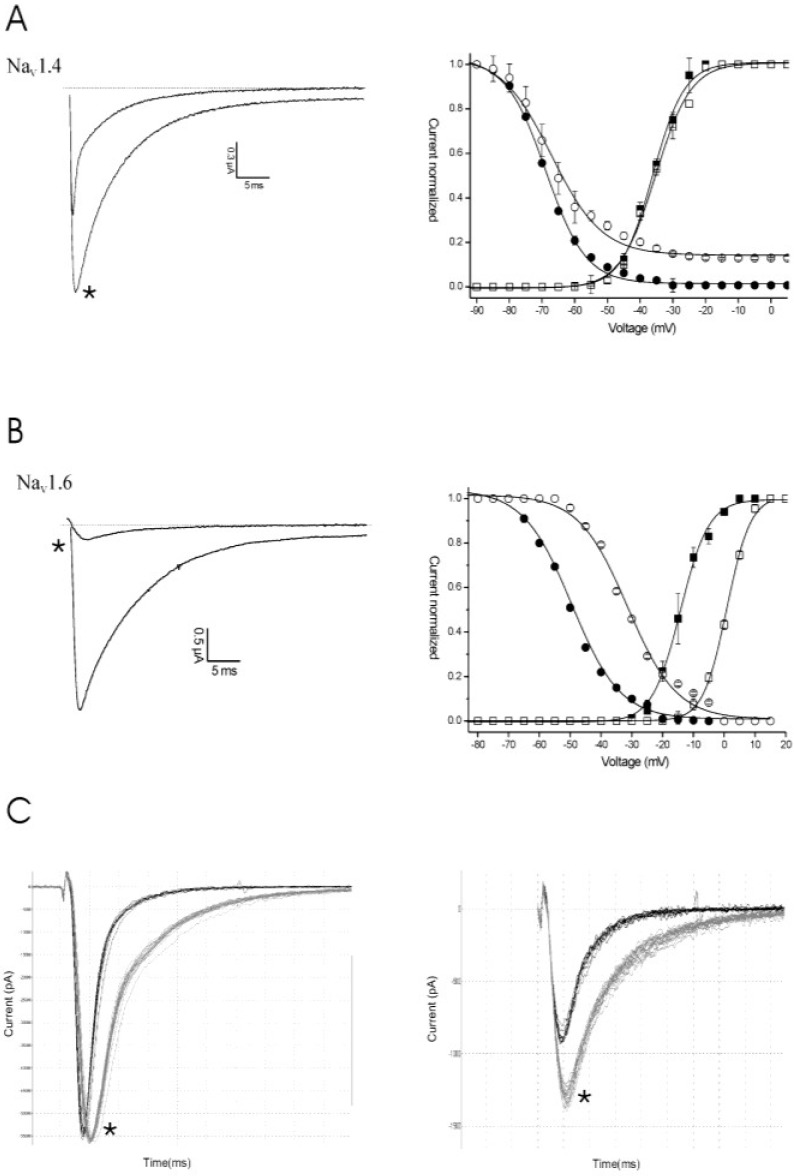
Electrophysiological characterization of Compound **8** on Na_V_1.4 (**A**,**C**) and Na_V_1.6 (**B**) channels under voltage clamp (**A**,**B**) and patch clamp (**C**) conditions. (**A**, left panel) Representative whole-cell current traces of Na_V_1.4 in control and 10 μM of Compound **8** conditions are shown; (right panel) steady-state activation and inactivation curves in control (closed symbols) and compound conditions (open symbols). (**B**, left panel) Representative whole-cell current traces of Na_V_1.6 in control and 250 μM of Compound **8** conditions are shown; (right panel) steady-state activation and inactivation curves in control (closed symbols) and compound conditions (open symbols). (**C**) Representative current traces of Na_V_1.4 in the control and 10 μM of compound conditions are shown in the resting state (left panel) and the inactivated state (right panel). The asterisk (*) marks the steady-state current traces after the application of 10 μM of Compound **8**.

Clathrodin, oroidin, hymenidin and their synthetic analogues (Figure 1) were subjected to testing against a broad panel of voltage-gated sodium channels. Electrophysiological experiments were performed both under voltage clamp conditions, using *Xenopus laevis* oocytes, as well as under patch clamp conditions, using Chinese hamster ovary [9] cells. In both expression systems, no activity was observed for clathrodin, oroidin, hymenidin or the analogues, **4**, **5**, **6** and **7**, even at concentrations up to 10 μM (Figure 2). However, the synthetic analogues, **8** and **9**, did show activity against several Na_V_ channel isoform subtypes. The application of 10 μM of Compound **8** resulted in an increase of the peak current of Na_V_1.2 and Na_V_1.4–Na_V_1.6, but not of Na_V_1.3, Na_V_1.7, Na_V_1.8 and the insect channel, BgNa_V_1.1 (Figure 3). Furthermore, a slowing down of the inactivation was observed for Na_V_1.2 and Na_V_1.4–Nav1.6 channels after the addition of **8**. The midpoint of the steady-state inactivation curve of Na_V_1.4 channels shifted non-significantly from −68.4 ± 0.3 mV in control conditions to −67.3 ± 0.9 mV after application of 10 μM of Compound **8**, while no significant alteration of the V_1/2_ of activation was noted (Figure 4a, left panel). When applying a high concentrations (>200 μM) of Compound **8**, a decrease of the sodium current peak amplitude could be observed. A 250 μM concentration of **8** decreased the current peak amplitude of Na_V_1.6 channels by 94.6% ± 2.2% (*n* = 4). The activation curves shifted significantly from −14.2 ± 0.3 mV in control conditions towards 0.9 ± 0.1 mV in the presence of Compound **8**. The midpoint of the inactivation curves shifted from −49.5 ± 0.9 mV in control conditions to −31.3 ± 0.5 mV (Figure 4b, left panel). These results were in concordance with the data obtained from the patch clamp experiments. Analysis of the QPatch experiments showed that 10 μM (**8**) caused a slowing down of the inactivation of the channels, both in the resting state (Figure 4c, right panel) and in the inactivated state (Figure 4c, left panel). Furthermore, in concordance with the voltage-clamp experiments, an increase in the sodium current peak amplitude was observed in the inactivated state (Figure 4c, left panel).

**Figure 5:**
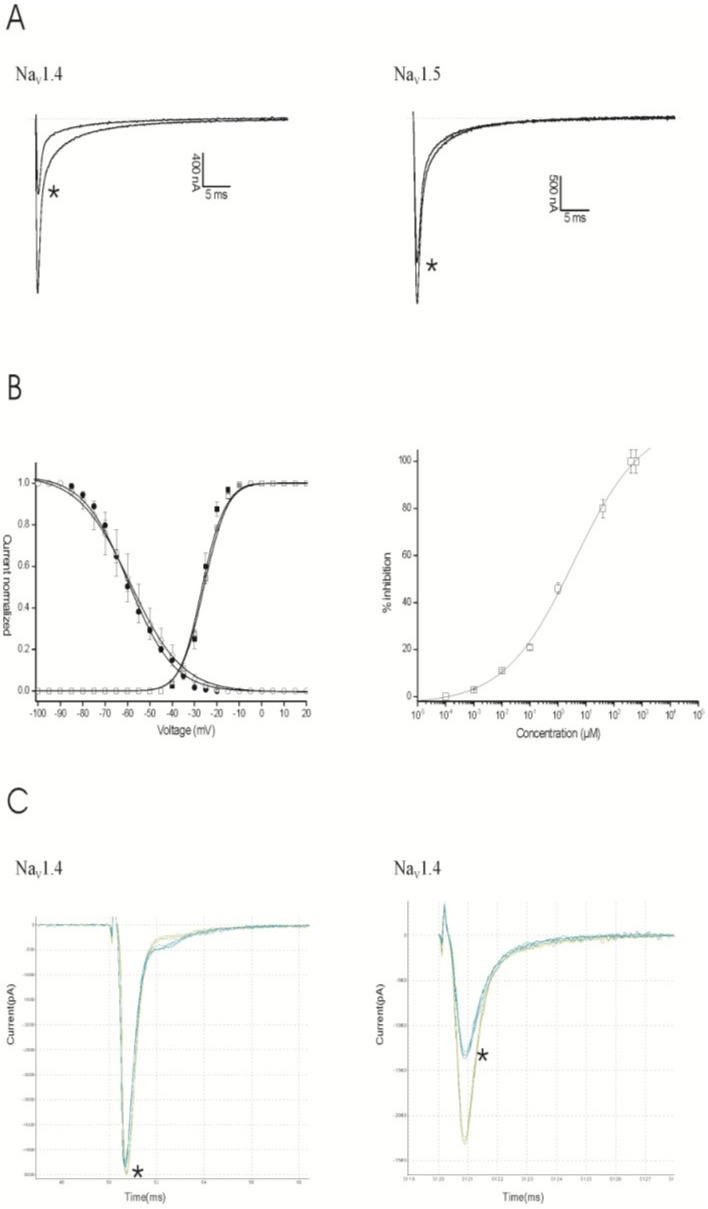
Electrophysiological characterization of Compound **9** under voltage clamp (**A**) and patch clamp (**C**) conditions. (**A**) Representative whole-cell current traces of Na_V_1.4 and Na_V_1.5 in the control and 1 μM of Compound **9** conditions are shown. The asterisk (*) marks the steady-state current traces after the application of 1 μM of Compound **9**. (**B**, left panel) At a 1-μM concentration of Compound **9**, no significant alteration in the characteristics of activation or inactivation was observed; (**B**, right panel) The concentration-response curve of Compound **9** for Na_V_1.4 channels. The IC_50_ value yielded 3.5 ± 0.9 μM. (**C**) The representative current traces of Na_V_1.4 in the control and 10 μM of Compound **9** conditions are shown in the resting state (left panel) and the inactivated state (right panel). The asterisk (*) marks the steady-state current traces after the application of 10 μM of Compound **9**.

The screening of Compound **9** indicated that this compound, at a concentration of 1 μM, is capable of inhibiting the current through Na_V_1.4 and Na_V_1.5 channels (Figure 5a). Compound **9** did not show a significant affinity for the other Na_V_ channel isoforms tested. The application of 1 μM of **9** inhibited 46.3% ± 2.4% and 19.6% ± 1.3% of the Na_V_1.4 (*n* = 5) and Na_V_1.5 (*n* = 5) channels, respectively. Cells heterologously expressing Nav1.4 channels were used to investigate if the observed inhibition results from a physical blocking of the pore or rather from a modulation of the kinetics of channel gating. At a 1-μM concentration of Compound **9**, no significant alteration in the characteristics of activation or inactivation was observed (Figure 5b, left panel). A concentration-response curve was constructed in order to assess the affinity of Compound **9** for Na_V_1.4 channels. The IC_50_ value yielded 3.5 ± 0.9 μM (Figure 5b, right panel). QPatch data showed that 10 μM of Compound **9** inhibits 40% of the sodium current through Na_V_1.4 channels in the inactivated state, but not in the resting state (Figure 5c).

### 2.2. Discussion

It has been well recognized that Na_V_ channels play a crucial role in inherited diseases, such as cardiovascular arrhythmias, central nervous system disorders and pain syndromes. This knowledge highlights Na_V_ channel isoforms as targets of novel compounds that will hopefully fulfil the unmet therapeutic need to successfully treat these disorders [8,10]. Therefore, small molecules capable of selective targeting and modulation of Na_V_ channel isoforms represent attractive pharmacological tools, either to identify the specific isoform involved in different channelopathies or as potential therapeutics. Over the last few decades, a number of compounds with promising pharmacological activity have been characterized from marine organisms. Several of these compounds have entered clinical trials or have been approved as drugs [11,12]. The marine alkaloid, clathrodin, isolated from the sponge, *Agelas clathrodes*, was reported to interact with Na_V_ channels. Patch clamp experiments on cells isolated from sympathetic ganglia of chick embryo indicated that clathrodin decreased the average maximum amplitude of inward sodium currents by approximately 30%, while shifting the voltage dependence of inactivation towards more negative potentials. Furthermore, voltage clamp experiments, using frog muscle, confirmed these results, since an early potentiation followed by complete inhibition of sodium currents was observed after the application of clathrodin. Surprisingly, in our hands, when testing clathrodin, oroidin, hymenidin and their analogues, Analogues **4**–**6**, no activity was observed, neither under voltage clamp nor patch clamp conditions. The lack of activity could be explained by different experimental conditions, such as different cells to express channels, potential differences in the posttranslational modifications of channels and differences in the expression of auxiliary subunits. Furthermore, the need for multiple pharmacological manipulations to isolate a specific type of channel current in tissue preparations, which are unnecessary in *Xenopus* oocytes or mammalian cells, and differences in membrane composition together with inherent differences in the osmolarity of the recording solutions, can provide an explanation for the differences observed between our experiments and earlier reports [5]. Nevertheless, from the experiments done in this work, it can be concluded that clathrodin does not interact with human Na_V_ channels and, therefore, is not further considered as a possible lead for the development of novel therapeutics targeting Na_V_ channels.

Based upon the clathrodin structure, a series of analogues have been synthesized and tested on the modulation of Na_V_ channels. Interestingly, one structural family of analogues (including Compounds **8** and **9**), in which a phenyl group was used to constrain the conformational freedom in the linker between the 2-aminoimidazole and indole cores, did show activity on Na_V_ channels. Both Compounds **8** and **9** showed Na_V_ channel activity, although it seems that they interact differently with the channels. At lower concentrations, Compound **8** alters the voltage dependence of steady-state inactivation, causing a slowing down of the inactivation of Na_V_ channels, which results in sustained non-inactivating currents. This effect is typically observed for certain scorpion toxins acting on Na_V_ channels (α-NaScTx) [13]. These peptide toxins bind at the so-called neurotoxin binding Site 3. This site is mainly located at the extracellular loops between the S3 and S4 segments of Domain IV of the sodium channel. α-NaScTx interacts with the channel in the closed state, stabilizing the S4 voltage sensor of Domain IV in its inward position and, thereby, impairing the conformational changes necessary for fast inactivation [14]. However, Compound **8** differs from α-NaScTx in its characteristics of channel modulation when higher concentrations of the compound are applied. At higher concentrations, a decrease of the sodium current peak amplitude could be observed. Whether this inhibition resulted from the strong shift in the V_1/2_ of both the activation and inactivation curves or rather from a reduction of the sodium conductance remains to be elucidated. Other alkaloids acting on Na_V_ channels, such as batrachotoxin (BTX), veratridine (VTD), aconitine and grayanotoxins, are also capable of reducing the sodium conductance. However, with BTX, this reduction is accompanied by several changes in channel properties, which are not seen upon the application of Compound **8**. When binding at neurotoxin Site 2, BTX will: (i) shift the voltage-dependence of activation toward a more negative potential; (ii) cause the inactivation to be slowed down or inhibited, resulting in sustained, non-inactivating currents; (iii) cause the sodium conductance through toxin-bound channels to be reduced; and (iv) cause the ion selectivity of modified channels to be altered due to a decreased discrimination for permeating ions [15,16]. Further structure-function studies, together with binding experiments, are required to verify which neurotoxin site is targeted by Compound **8**.

For Compound **9**, the inhibition of channels without an alteration of gating kinetics was observed, suggesting that the inhibition results from a physical obstruction of the sodium ion pathway. Tetrodotoxin (TTX) and saxitoxin (STX) are two marine alkaloids known to inhibit Na_V_ channels. STX and TTX are the most studied and well characterized members of the large family of guanidinium-containing marine compounds. Even though STX and TTX and their respective analogues belong to structurally divergent families, they do bind with high affinity to the same target, the neurotoxin receptor Site 1 of Na_V_ channels [14,17]. Upon binding at a narrow part within the channel pore, they obstruct the ion conductance through the channels by physically blocking the pathway of the Na^+^ ions. Similar to Compound **9**, neither toxin significantly alters the kinetics of gating. Although Compound **9** exerts a similar pharmacological activity upon binding to Na_V_ channels, it should be noted that it is structurally unrelated to the guanidinium toxins TTX and STX. Therefore, further structure-function studies combined with site-directed mutagenesis experiments are required to verify whether or not Compound **9** interacts with the neurotoxin receptor Site 1 of Na_V_ channels.

## 3. Experimental Section

### 3.1. Compound Synthesis

Clathrodin (**1**), oroidin (**2**) and hymenidin (**3**) were synthesized by a modified procedure of Al-Mourabit *et al.* [18]. Dihydroclathrodin (**4**) was obtained by acylation of 2-amino-4-(3-aminopropyl)imidazole prepared from l-ornithine by the improved synthesis of Olofson *et al.* [19,20]. Compounds **5**, **6** and **7** were prepared by acylation of 3-amino-1-(2-aminoimidazol-4-yl)prop-1-ene [20]. Compounds **8** and **9** were prepared by coupling of tert-butyl 2-amino-4-(3-aminophenyl)-1*H*-imidazole-1-carboxylate and indole-2- or indole-3-carboxylic acid, as described [21]. The purity of the tested compounds was established to be >95% by HPLC analysis (Agilent Technologies HP 1100 instrument with a G1365B UV-Vis detector, using an Agilent Eclipse plus C18 column (4.6 × 150 mm, Technologies, Santa Clara, CA, USA) and a mixture of 0.1% NH_3_ in water (A) and methanol (B) as the eluent. The gradient was 10% B to 70% in 20 min at a flow rate 1 mL/min).

### 3.2. Electrophysiology

Patch clamp experiments: Cells were prepared by dissociation from T175 cell culture flasks using trypsin-EDTA (0.05%). Cells were kept in serum-free media in the cell hotel on-board the QPatch HT. These cells were sampled, washed and re-suspended in extracellular recording solution by the QPatch HT immediately before the application to the well site on the chip. Once in whole-cell configuration, the vehicle (0.1% DMSO v/v) was applied to the cells to achieve a stable control recording (4 min total). This was followed by the application of test concentrations as a single bolus addition (4-min incubation per test concentration). Compounds were prepared in extracellular recording solution from a 10-mM (100% DMSO) stock to yield a final 10-μM (0.1% DMSO) test concentration from which subsequent serial dilutions in extracellular solution were performed (0.3–10 μM). Currents were elicited from Nav1.3, Nav1.4 and Nav1.7 cell lines using a standard two-pulse voltage protocol. From a holding potential of −100 mV; a 20-ms activating step to −20 mV was applied to assess the effect of compounds on the resting (closed) state block. The second activating pulse was applied following a 5-second pre-pulse to half inactivation potential (variable, depending on the sodium channel studied, −65 to −75 mV) to assess the block on the open-inactivated state of the channel. This protocol was applied at a sweep interval of 0.067 Hz throughout the duration of the experiment. To study Na_V_1.5 currents, a pulse train consisting of 10 repetitive activating test pulses to −20 mV from a holding potential of −100 mV were applied at a 1-Hz frequency until 10 pulses were reached; this sequence was repeated at a sweep interval of 0.016 Hz throughout the duration of the experiment. For Na_V_1.3, Na_V_1.4 and Na_V_1.7 channels, the peak inward current was determined for both the closed and open-inactivated test pulses from each sweep applied to the cells and for Na_V_1.5 from the tenth pulse of each pulse train recorded. Data was captured using QPatch assay software (v5.0). The percent of inhibition of the peak current was calculated as the mean peak current value for the last three sweeps measured in each concentration test period relative to the last three sweeps recorded during the control vehicle period. Sigmoidal concentration response curves (four parameter logistic curves) were fitted to the percent of inhibition data using Xlfit (IDBS), from which the IC_50_ was determined. Fits were constrained at 0% and 100%. Data are presented as the mean ± SD for a minimum of 3 independent observations.

### 3.3. Two-Electrode Voltage Clamp

#### 3.3.1. Heterologous Expression

For expression in *X. laevis* oocytes, Na_V_1.3/pLCT2 (NotI), Na_V_1.4/pUI-2 (NotI), Na_V_1.7/pBSTA.rPN1 (SacII) and hβ1/pGEM-HE (NheI) were linearized with the respective restriction enzymes, mentioned between parentheses, and transcribed using the T7 mMESSAGE-mMACHINE transcription kit (Ambion, Austin, TX, USA). hNa_V_1.5/pcDNA3.1 (XbaI) was linearized with the respective restriction enzyme, mentioned between parentheses, and transcribed with the SP6 mMESSAGE-mMACHINE transcription kit (Ambion, Austin, TX, USA). Stages V and VI oocytes, harvested from anesthetized female *X. laevis* frogs, as described previously [22], were injected with 30–50 nL of 1–3 μg/μL Na_V _channel cRNA using a micro-injector (Drummond Scientific, Broomall, PA, USA). The oocytes were then incubated in ND96 solution (in mM: NaCl 96, KCl 2, MgCl_2_ 1, CaCl_2_ 1.8, HEPES 5), adjusted to pH 7.5 and supplemented with 50 mg/L of gentamycin sulphate and 90 mg/L theophylline, at 16 °C for 1–5 days, until the expression of ion channels.

#### 3.3.2. Electrophysiological Characterization

Two-electrode voltage-clamp (TEVC) recordings were performed at room temperature using a GeneClamp 500 amplifier (Molecular Devices, Sunnyvale, CA, USA) controlled by a pClamp data acquisition system (Molecular Devices). Whole-cell currents from oocytes were recorded 1–5 days after injection. Voltage and current electrodes were filled with 3 M KCl. The resistances of both electrodes were kept between 0.7 and 1.7 MΩ. The elicited currents were sampled at 20 kHz and filtered at 2 kHz using a four-pole, low-pass Bessel filter. To eliminate the effect of the voltage drop across the bath-grounding electrode, a two-electrode bath clamp actively controlled the bath potential. Leak subtraction was performed using a −P/4 protocol. For the electrophysiological characterization of the compounds, a number of voltage protocols were applied from a holding potential of −90 mV with a start-to-start pulse frequency of 0.2 Hz. Current traces were evoked in oocytes expressing the cloned Na_V_ channels according to the following protocol: from a holding potential of −90 mV, current traces were evoked by 100-ms depolarizations to the voltage corresponding to the maximal activation of the Na_V_ channel subtype in control conditions. All data were tested for normality using a D’Agustino Pearson omnibus normality test. Data following a Gaussian distribution were analysed for significance using one-way ANOVA and the Bonferroni test. Non-parametric data were analysed for significance using the Kruskal-Wallis and Dunn’s test.

## 4. Conclusions

There is increasing evidence not only regarding the important role of Na_V_ channels in channelopathies, but also their crucial contribution in chronic pain. Consequently, there is an urgent need for novel therapeutics targeting Na_V_ channels. Based on previous work, we reinvestigated whether or not clathrodin and its analogues are suitable as leads in the development of novel therapeutics exerting a pharmacological activity through an interaction with Na_V_ channels. In this work, we show that clathrodin does not interact with Na_V_ channels. However, two analogues of clathrodin, Compound **8** and **9**, are capable of modulating Na_V_ channels. Unfortunately, these compounds showed affinity for a broad range of Na_V_ channels with a low potency. Nevertheless, these compounds might serve as templates for the development of more selective and potent compounds in future studies.
